# Conducting Polymers in the Design of Biosensors and Biofuel Cells

**DOI:** 10.3390/polym13010049

**Published:** 2020-12-25

**Authors:** Simonas Ramanavicius, Arunas Ramanavicius

**Affiliations:** Department of Physical Chemistry, Institute of Chemistry, Faculty of Chemistry and Geosciences, Vilnius University, Naugarduko 24, LT-03225 Vilnius, Lithuania

**Keywords:** conducting polymers (CPs), biosensors, microbial and enzymatic biofuel cells, immunosensors, glucose biosensors, polymer-modified electrodes, electrochemical deposition, electrochemical sensors, bioelectrochemistry, electrochromic organic polymers

## Abstract

Fast and sensitive determination of biologically active compounds is very important in biomedical diagnostics, the food and beverage industry, and environmental analysis. In this review, the most promising directions in analytical application of conducting polymers (CPs) are outlined. Up to now polyaniline, polypyrrole, polythiophene, and poly(3,4-ethylenedioxythiophene) are the most frequently used CPs in the design of sensors and biosensors; therefore, in this review, main attention is paid to these conducting polymers. The most popular polymerization methods applied for the formation of conducting polymer layers are discussed. The applicability of polypyrrole-based functional layers in the design of electrochemical biosensors and biofuel cells is highlighted. Some signal transduction mechanisms in CP-based sensors and biosensors are discussed. Biocompatibility-related aspects of some conducting polymers are overviewed and some insights into the application of CP-based coatings for the design of implantable sensors and biofuel cells are addressed. New trends and perspectives in the development of sensors based on CPs and their composites with other materials are discussed.

## 1. Introduction

Demands for fast biomedical, environmental, and food and beverage analysis are evolving very rapidly. Therefore, various new and advanced technologies are required to fulfil new trends and requirements of analytical systems. Recently, the determination of many biologically active materials in various samples has been performed by sensor- and biosensor-based techniques [1], which are finding applications in the solution of challenging problems of pharmaceutical and biomedical analysis [2,3]. In order to extend the bioanalytical applicability of sensors, many types of semiconducting materials [4], polymers, and polymer-based composites are used. In this respect, conducting polymers are among the most promising materials that can be applied in order to extend the analytical characteristics of sensors [5]. Electrochemical activity, electrical conductivity, mechanical elasticity, biocompatibility, and environmental stability of conducting polymers [6] are among the most desired properties required for the advancement of sensing performance of analytical and bioanalytical systems. In addition, some conducting polymers possess very good electrical conductivity [7,8] and unique capabilities to transfer electric charge from redox enzymes towards electrodes [9].

Among a variety of CPs, polypyrrole (Ppy), polyaniline (PANI), polythiophene (PTH), and poly(3,4-ethylenedioxythiophene) (PEDOT) are mostly applied because of their high technological potential, which has been exploited not only in sensors [10,11,12,13] and biosensors [14], but also in the design of rechargeable batteries [15], corrosion preventing coatings [16], electromagnetic shielding [17], solar cells [18], and super capacitors [19,20,21]. Many studies have demonstrated that conducting polymers might be synthesized by electrochemical [14,22], chemical [23], and even biotechnological approaches [5]. After the synthesis/deposition, conducting polymer-based layers can be very easily doped and de-doped by electrochemical techniques; this doping-procedure enables to tailor the properties of the conducting polymer-based sensing layer. Moreover, from the technological point of view, it is very attractive that different conducting polymer-based composite materials can be modified by entrapped enzymes [24], receptor-like proteins [14], antibodies [25], and DNA [26] in order to advance their sensitivity and selectivity. Therefore, such conducting polymer-based composite materials can show unique sensing properties, which are determined by entrapped materials and/or dopants. However, the main disadvantages of sensing devices based on biological materials are limited stability and rather high costs. To solve these problems, CP-based molecularly imprinted polymer-based sensors were developed, which possess selectivity and sensitivity almost the same as that of biosensors based on the application of some biological materials [27].

Conducting polymers offer a variety of technological solutions required for the development of electrochemical biosensors. The aim of this review article is to overview major methods of synthesis and bioanalytical application of some conjugated polymers including polypyrrole, polytiophene, polyaniline, and PEDOT, among others. The applicability of conducting polymers in the design of biosensors is critically overviewed, discussed, and evaluated. Some new insights into the application of CPs in the design of biofuel cells are critically discussed.

## 2. Formation of Conducting Polymer-Based Sensing Structures

Many different conducting polymer synthesis methods were developed recently, most of them based on electrochemical, chemical, and biochemical formation of conducting polymers (CPs). Some the methods with the best prospects for the formation of CPs are overviewed, outlined, and discussed in this chapter. Most of the attention has been paid to these methods, which were applied and/or developed by some authors of this review. Selection of the most appropriate monomer for the formation of the sensing CP layer is a critical issue in the development of any kind of sensor.

Chemical synthesis based on oxidizing compounds is one of the most popular methods to form conducting polymer-based sensing structures. This method is simple and is based on the application of strong oxidants such as FeCl_3_ or H_2_O_2_ [23]. In some of our studies, we have demonstrated that spherical particles of polypyrrole can be formed using H_2_O_2_, which has oxidation potential sufficient to initiate the polymerization of pyrrole and some other monomers, which form conducting polymers. This method is very attractive because the excess of H_2_O_2_ degrades into H_2_O and O_2_; in such a way, very pure CPs particles can be synthesized. Our studies have demonstrated that such particles possesses a good biocompatibility with living stem cells [28,29] and with the immune system of mice [30]. Very low irritation of the immune system was confirmed by the injection of Ppy-based nano- and micro-particles into mice peritoneum [30]. The advantage of such oxidative-chemical synthesis is that, using this method, large quantities of nanoparticles and/or CP-based composite materials, which are suspended in a solution and/or deposited on selected surface, can be formed. During the next development steps, the formed nanoparticles can be modified by different methods. However, such a method of CP development is not always suitable for the formation of polymer-based films, which are required for sensor design. In our research, we have shown that Ppy can be synthesized by redox cycling of [Fe(CN)_6_]^4−^/[Fe(CN)_6_]^3−^, which is suitable for the formation of polypyrrole [31].

**Figure 1:**
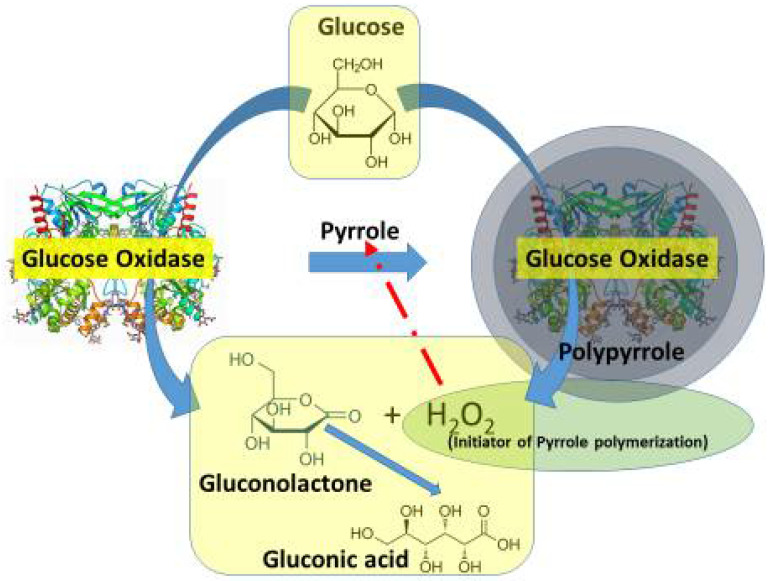
Enzymatic formation of polypyrrole around enzyme glucose oxidase, which, during catalytic action, generates H_2_O_2_ that acts as an initiator of pyrrole polymerization reaction.

Enzymatic synthesis of conducing polymers. Enzymatic methods for the formation of CPs are based on the application of oxidoreductases and/or redox compounds formed by these enzymes [32]. These ‘green’ synthesis-based reactions are performed in environmentally friendly conditions, room temperature, and pHs [33]. Redox enzymes can act as oxidizers or, during the enzymatic reaction, can create some strong oxidants (e.g., H_2_O_2_), which are required for the synthesis of CPs [34,35] (Figure 1). Enzymatic synthesis of CPs can be performed using redox enzymes (e.g., glucose oxidase and many other oxidases), which, during catalytic action, generate hydrogen peroxide [5]. Hence, glucose oxidase (GOx) from *Penicillium vitale* can be used for the formation of various conducting polymers, including the following: polypyrrole [5,14,36], polyaniline [37], polytiophene [38], polyphenanthroline [9], poly-9,10-phenanthrenequinone [39], and some other conducting polymer-based layers and nanoparticles. This is a very useful method, which is based on the oxidation of polymerizable monomers by H_2_O_2_ formed during catalytic action of GOx. Both (i) immobilized and (ii) dissolved in water GOx have been successfully applied in the enzymatic synthesis of conducting polymer-based layers and/or particles with entrapped enzymes, which produce hydrogen peroxide. The enzymatic synthesis of CPs is interesting because of good compatibility of formed structures with enzymes [40,41]. It is very useful that entrapped GOx retains catalytic activity while being encapsulated within such particles and/or layers, which is well applicable for the design of amperometric biosensors and biofuel cells.

Microbiological synthesis of polypyrrole. The application of microorganisms in the synthesis of conducting polymers offers even more advantages in comparison with the application of isolated enzymes, because microorganisms can retain their activity during the long-lasting life cycle of single microorganism and/or culture based on high number of micoorganisms [42]. Hence, microorganisms have been used for the synthesis of some polymers [43]. Recently, our research team applied whole microorganisms for the synthesis of conducting polymer polypyrrole. In the initial research, which has reported bacteria-assisted synthesis of polypyrrole, we used bacterial cells *Streptomyces* spp., which produce some phenol-oxidases; therefore, these enzymes are able to perform polymerization of pyrrole by forming Ppy-based hollow Ppy microspheres [44]. In another research, we applied living cell induced redox cycling of [Fe(CN)_6_]^4−^/[Fe(CN)_6_]^3−^, which enabled the formation of polypyrrole within the yeast cell wall [45]. We demonstrated that these kinds of Ppy formation redox processes, which are involved in metabolism occurring in living cells, can be adapted even without any redox mediators [46]. Our studies based on the nonradioactive isotope method illustrated that Ppy formed during microbial polymerization is deposited mainly within the cell wall and in the space between the cell wall and cell membrane [47].

It is interesting that, during the microbial synthesis of conducting polymers, cells retain sufficient viability, and the formed Ppy is integrated within the cell wall and in the periplasmic area of the microorganism. The formation of Ppy is induced by the action of redox enzymes, which are present in living cells; therefore, CP-based clusters are growing in close proximity to the cell membrane and within the cell wall; furthermore, the formed Ppy changes the elasticity and dielectric permittivity of the cell wall [47]. For Ppy-modified yeast cells, it was determined that some Ppy structures are formed within the cell membrane, but the highest concentration of Ppy is formed within the periplasm and/or within the cell wall of living cells [47]. During this process, sufficient conductivity of some conducting polymers was achieved, which enables the enhancement of charge transfer from some microorganisms such as *Rhizoctania* sp. and *Aspergillus niger* [46,48,49], and is useful for the development of biofuel cells [46]. Some other authors also proved this approach and applied it for the modification of several different bacteria, namely, for *Streptococcus thermophilus*, *Ochrobacterium anthropic*, *Shewanella oneidensis*, and *Escherichia coli* [50], which exhibited both sufficient viability and advanced cell wall conductivity. Owing to advanced cell wall conductivity, such microorganisms became suitable for the design of new microbial sensors [51,52] and microbial fuel cells (MBFCs) [46].

The same polymerization principle was applied for the modification of mammalian cell lines [53] using redox cycling of [Fe(CN)_6_]^4−^/[Fe(CN)_6_]^3−^, which is suitable for the formation of polypyrrole in solution and [31] within living cells [45]. Therefore, it is expected that such CP-modified mammalian cell lines will find some particular applications in biofuel cells and in some other bioelectronics-based devices.

Electrochemical deposition. Unfortunately, most CPs have rather bad solubility in the most of the known solvents; therefore, low solubility reduces the processability of formed CPs. For this reason, electrochemical deposition seems very useful for the deposition of conducting polymer-based layers directly on electrodes and on some other conducting surfaces. The variation of electrochemical parameters including potential, current, potential sweep rate, and duration enables one to tune the analytical characteristics of the formed polymeric layers [14,54]. In addition, electrical conductivity and some electrochemical properties of conducting polymers can be tailored and controlled by the variation of polymerizable monomer concentrations, pH of polymerization bulk solution, and applying different dopant concentrations [55,56,57]. Hence, the morphology and thickness of the formed layer can be controlled by the adjustment of the potential-profile [14] and pH of polymerization bulk solution [58]. Morphology control enables one to improve and even to tune the performance of the electrochemically formed CP-based layer [59].

Therefore, electrochemical formation of conducting polymer-based layers is very attractive because CP-based composite layers with different sensitivity and selectivity can be formed. Electrochemical formation of CP layers enables one to form the layers with significant structural differences, which generate different electrochemical responses towards different analytes; therefore, such structures can be applied in the design of electrochemical arrays, which can be characterized by different response patterns, which can be analyzed using multivariate analysis of variance (MANOVA) [60]. The application of CP fluorescent-based sensors enables one to exploit both (i) the photoluminescence of formed structures [61] and (ii) photoluminescence quenching by conducting polymers based on the Forster resonance energy transfer (FRET) mechanism, which, at low distances, induces the quenching of photoluminescence emissions, in order to decrease the influence of nonspecific interaction of other proteins with the immunosensor based on immobilized proteins [62,63]. Some conformational and morphological changes are exploited to manipulate the duration of the exciton life period, which is also reflected in photoluminescence signal [64]. The quenching efficiency of CPs, like many other photoluminescence quenchers, is distance-dependent; therefore, CPs are well suited for the quenching of photoluminescence of non-specifically adsorbed proteins and other photoluminescent structures, which non-specifically adsorb directly on the signal transducer, while on the immobilized receptor, bonded analytes appear out of this rather short distance, which is required for the quenching of photoluminescence [62,63].

Some CPs were applied in simple optical/visual [65,66] and photoluminescence-based [67] sensing, e.g., a conjugated microporous polymer was applied as a sensing platform for the determination of aminoglycoside antibiotic in water [68]. CPs based on carbazole-based structures (namely, *N*-benzyldibromo-carbazole, *N*-benzyldimethoxy-carbazole, and *N*-benzylcarbazole) were applied in the design of fluorescence-based sensors and were applied for the determination of some pesticide (glyphosate, trifluralin, cyfluothrin, fenitrothion, imidacloprid, and isopropalin) concentrations [69].

Diffusion of analyte (in the case of biosensors) and organic fuel (in the case of biofuel cells) by other compounds through the CP-based matrix is also a very important issue for the efficient performance of these structures, as it is responsible for the limits in electrical current generation. In some cases, a three-dimensional CP-based network can be made very porous, which enables to increase the permeability and even electrical capacitance of CP-based films [70]. Most CPs are deposited under kinetic control and, therefore, are amorphous and the formed layers do not have a long-range molecular order. The possibility to tune the micro-porosity and, in a such way, to change the effective surface area using organic molecules as ‘connectors’ between different molecules, enabled the achievement of an ordered and porous conjugated structure of CP [71].

## 3. Chemical and Physical Properties of Conducting Polymers

CPs contain delocalized π-electrons within the backbone of the polymeric chain; therefore, these polymers possess advanced electrical conductivity and some other unique properties, such as low ionization potential [58,72,73]. Therefore, conducting polymers (CPs) have found efficient applications in sensors, rechargeable batteries, electrochromic displays, transistors, photovoltaic devices, some light emitting diodes and smart windows [74,75,76]. The most important issue in the design of electronic devices is the selection of CPs with suitable properties [77,78]. In some studies, it was demonstrated that a conducting polymer, polypyrrole, exhibits unique electrical, electrochemical [5], affinity [79], and/or optical [80] properties. Therefore, changes in one or more of these physicochemical properties of the CP-based biological recognition layer (e.g., impedance, variation of electrical capacitance, changes of optical or photoluminescence properties) can be determined/monitored by a particular signal transducing system. Among the high number of CPs, polypyrrole has been used mostly in the design of enzymatic biosensors as a matrix for enzyme immobilization [5]. Some CPs form hydrogels [81], which consist of two phases, (i) a liquid phase and (ii) a ‘solid’ CP-based phase. Therefore, they are well suited for the entrapment of biomolecules, which retains their functionality practically only in a water-based environment. In addition, the porous structure of gels enables good diffusion of analyte molecules and ions through a gel-based structure [82,83]. Conducting polymer-based nanocomposites (EDTA-PANI/SWCNTs nanocomposite) show selectivity towards some heavy metal ions, thus they are well suited for the design of sensors dedicated to the determination of copper (II) [84,85], lead (II) [85], mercury (II) [85], and many other metal ions [86].

It was demonstrated that some CP-based structures can exhibit real multi-functionality, because they can be applied not only for sensing purposes, but also for the removal of some hazardous compounds from environmental samples [87].

## 4. Conducting Polymers for the Design of Enzymatic and Other Catalytic Biosensors

CPs have recently been applied in many types of catalytic sensors and biosensors that can be used for various bioanalytical purposes. Redox enzyme–glucose oxidase (GOx) is the most frequently applied in the design of biological recognition elements of glucose biosensors. As reported above, GOx can act as a biocatalyst in the formation of many conducting polymers including polypyrrole, polyaniline, and polytiophene.

**Figure 2:**
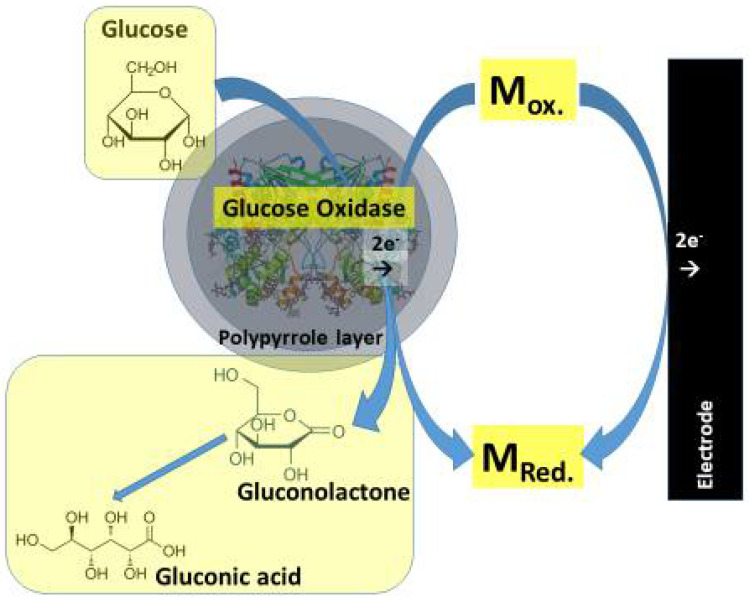
The application of polypyrrole modified glucose oxidase in the design of enzymatic sensor mediated by a redox mediator (M_Ox._/M_Red._) system. Phenazine methosulfate, benzoquinone, 2,6-dichlorophenol indophenol, and many others redox compounds can act as redox mediators.

Amperometric biosensors based on CPs. The Ppy layer has relatively low permeability towards both enzyme-substrates and formed reaction products; therefore, the apparent Michaelis constant (*K*_Mapp_) of enzymes immobilized within conducting polymers significantly increases for all substrates. This feature extends the ‘linear range’ of sensors, where enzymes are immobilized within the CP-based matrix [24]. Therefore, such an effect can be well exploited in the design of biosensors based on such enzymes, which are characterized by low *K*_Mapp_ and, for this reason, when immobilized without the formation of the ‘diffusion layer’, such biosensors are not well suited for the investigation of aliquots containing high concentrations of substrates; for example, the concentration of glucose in human blood serum is significantly higher than the *K*_Mapp_ of the most frequently used glucose oxidases [88]. Hence, the entrapment within CPs enables to tune the analytical characteristics of biosensors including detection limits and linear ranges [5,37,38]. The application of GOx modified by polypyrrole in the design of an enzymatic sensor mediated by a redox mediator (M_Ox._/M_Red._) system, where phenazine methosulfate, benzoquinone, 2,6-dichlorophenol indophenol, and many others redox compounds can act as redox mediators, is presented in Figure 2.

Some conducting polymers can be involved in the charge transfer between the electrode and redox active center of the enzyme [9] and, for this reason, they are very promising in the design of amperometric biosensors [24] biofuel cells and some other bioelectronic devices [5]. It should be noted, however, that most enzymes have redox-sites that are deeply embedded within the protein ‘shell’. For this reason, electron transfer from these redox sites is hardly possible even if they are immobilized within the CP-based matrix. In some of our studies, we have demonstrated that this problem can be solved by the grafting of some CPs (e.g., poly-phenontraline) to the surface of glassy carbon electrode [9]. GOx is the most frequently used enzyme in the design of glucose biosensors, which are used for biomedical purposes and as model systems during the development of enzymatic sensors. During the modelling of glucose sensors, GOx was immobilized within many different conducting polymers [24]. Charge transfer between the immobilized enzyme and the electrode is a critical problem in redox enzyme-based sensors; in most cases, this problem is solved by the application of soluble redox mediators, but in some cases, charge transfer can be established via an electrochemically formed/deposited CP network [9]. In such sensors, CPs play a dual role as the immobilization matrix and as the charge transfer chain [89,90]. In some cases, it is reasonable to apply copolymers, which contain some functional groups (e.g., carboxyl group) suitable for covalent immobilization of the enzyme on the surface of the formed CP-based copolymer layer, as shown by the development of the amperometric glucose biosensor based on poly(pyrrole-2-carboxylic acid)/glucose oxidase biocomposite [91]. During the development of amperometric enzymatic biosensors dedicated for continuous measurements, one of the major challenges is related to the instability of the analytical signal. Immobilized enzymes are inactive; the CP-based layer swells during continuous measurements; and, therefore, the analytical signal changes in time [92]. However, this important problem can be easily solved by periodical recalibration of such amperometric biosensors, which is applied after a certain number of measurements. However, the long life stability of enzymatic biosensors is still a challenge, which needs additional investigations.

**Figure 3:**
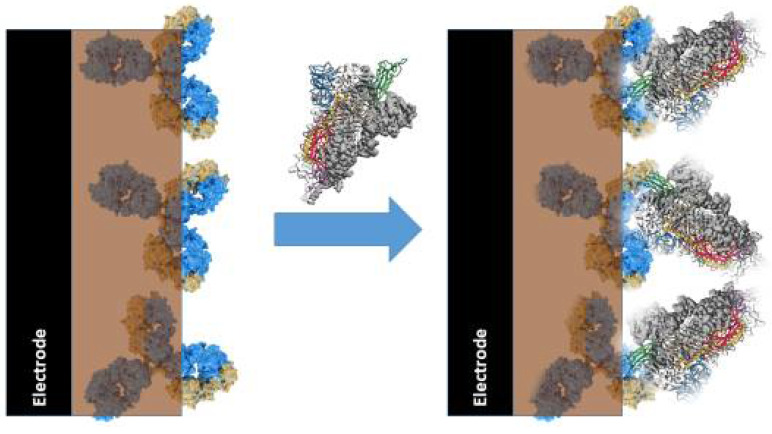
Action of immunosensor based on antibodies immobilized within conducting polymer, polypyrrole, and the formation of the immune complex between immobilized antibodies and analyte-protein (e.g., virus proteins) present in the dissolved aliquot.

**Figure 4:**
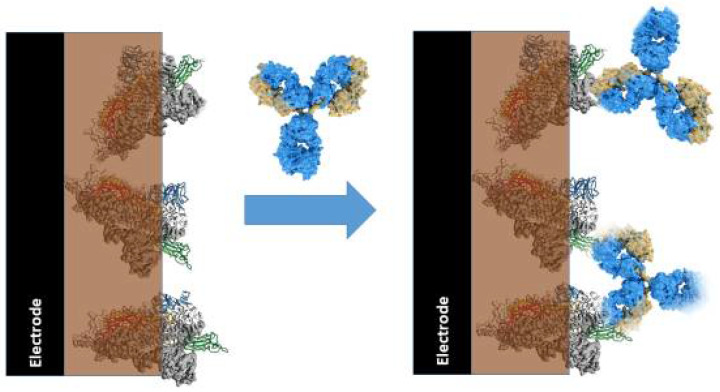
Action of immunosensor based on antigens (e.g., virus nucleocapside proteins) immobilized within conducting polymer, polypyrrole, and the formation of the immune complex between immobilized protein (e.g., virus proteins) and antibodies against these proteins, which are present in the dissolved aliquot.

Affinity biosensors based on conducting polymers. In affinity sensors, CPs can be applied as immobilization matrixes [14], signal transduction systems [9,14], and even analyte recognizing structures based on molecular imprints formed within deposited layers of CPs [79,93,94,95]. Electrochemically formed layers of CPs with entrapped specific recognition properties with proteins (e.g., receptors, antibodies (Figure 3), or antigens (Figure 4)) have been applied in the design of various types of immunosensors [14]. In some immunosenors, the CP-based structure enhances the electrochemical signal, which is generated by potentiodynamic methods [14]. In some of our studies, we demonstrated that polypyrrole-based matrix reduces the influence of some interfering materials when a photoluminescence-based analytical signal is registered [62]. The selection of proper immobilization methods is very important for the development of any kind of biosensor, because the application of suitable immobilization methods ensures appropriate performance of the designed bioanalytical system. However, this feature is especially important during the design of affinity sensors, because only proper orientation of receptors [96] and/or antibodies [97] enables efficient interaction with the analyte.

## 5. Molecularly Imprinted Polymers (MIPs) Based Sensors

Conventional bioanalytical techniques based on enzyme linked immuno sorbent assay (ELISA) are suitable for the accurate detection of target molecules, but biological materials applied in the design of ELISA kits are expensive and the analysis procedure itself is tedious and long-lasting. Therefore, a very promising alternative is the application of some affinity sensors. Among different affinity sensors (antibody-based immunosensors, natural- or artificial receptor-based sensors, DNA-aptamer-based sensors, MIP-based sensors, and so on), MIP-based sensors are very promising, because they are mainly based on the polymeric-matrix [98,99,100] and do not require biological recognition materials, which are usually very expensive. MIPs are polymers that have artificially created specific molecular recognition sites, which are complementary to the imprinted target molecule. Hence, MIPs mimic the action of receptors and antibodies. Polymers such as methacrylic acid, acrylamide, and acrylic acid are often used to design MIP-based sensors; such sensors are mostly suitable for the determination of low molecular weight analytes [101,102,103,104]. MIP based on methacrylic acid was applied in the design of sensors for the determination of six different steroids, namely, testosterone, Δ4-androstene-3,17-dione, 1,4-androstadiene-3,17-dione, β-estradiol, progesterone, and testosterone propionate [103], and a bifunctional monomer, N-phenylethylene diamine methacrylamide, was used for the construction of the electrochemical sensor for the detection of β-estradiol [104]. Some other researchers have adapted Fe_3_O_4_-based magnetic nanoparticles in order to design nanostructured magnetic molecularly imprinted polymers for 17-β-estradiol determination [105].

**Figure 5:**
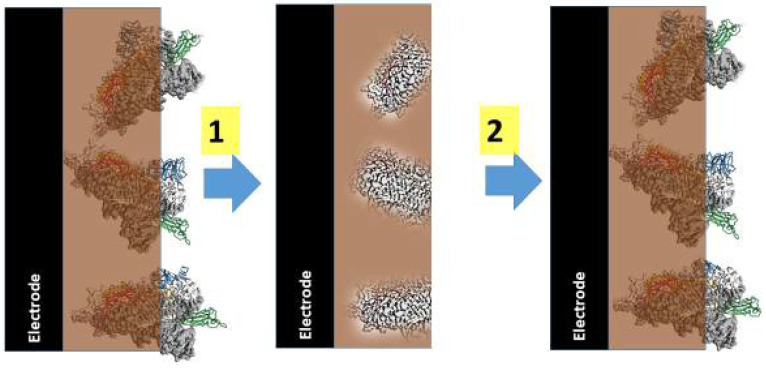
Preparation and action of the molecularly imprinted polymer (MIP)-based sensor based on imprinted virus proteins; 1—extraction of imprinted proteins and 2—action of MIP-based sensor in the solution containing similar proteins that were imprinted.

Electrochemical polymerization and chemical polymerization, which can be followed by overoxidation, can be applied for the design of MIPs based on CPs. Oxidative polymerization is very simple and cheap; therefore, very large quantities of MIPs can be produced by this method [106,107]. Electrochemical polymerization has some advantages over oxidative polymerization; because this method allows the formation of an MIP layer over the electrode or other conducting surface, it can be performed in different solvents dependending on the requirements for polymerizable monomer and/or imprinted analyte. Moreover, electrochemical polymerization enables to change the morphology, homogeneity, conductivity, thickness, and overoxidation level of the formed polymer layer with molecular imprints. There are many different MIP formation techniques, but all of them include several common stages. Most monomers with attached functional groups that are required for the recognition of target molecule are co-polymerized with cross-linking monomers, which do not have any recognition properties in the presence of the target molecule and, during the next stage, the target molecule is extracted from the formed MIP [108]. However, some authors have reported some problems during the extraction of imprinted target molecules [109]. Therefore, in the case of molecularly imprinted polypyrrole formation, the overoxidation can be applied as a solution to this problem. The development of MIPs based on overoxidized polypyrrole seems to be one of the most promising directions among the application of all CPs, because this polymer can be formed by a very simple electrochemical procedure using a single polymerizable monomer, pyrrole [110]. CPs can be well imprinted by low molecular weight molecules, such as uric acid [93], caffeine [10], theophylline [27,111], ganciclovir [112], tetracycline [110], dopamine [113], adrenaline [114], L-aspartic acid [115], testosterone [116], serotonin [117], and histamine [118]. It should be noted that Ppy can be imprinted even by large molecular weight biomolecules, such as DNA [29,79,119] by proteins [120,121,122,123,124] (Figure 5), by rather large spores (e.g., *bacillus cereus* spores) [125], or even by whole bacteria such as *Escherichia coli* [126].

However, PANI, because of its more rigid structure, is still rarely imprinted by some analytes, e.g., a sensor based on molecularly imprinted PANI for the determination rather low concentrations (below 0.1 nM) of antibiotic azithromycin [127] and of *bacillus cereus* spores [125] has been reported.

The application of the most appropriate monomer for the formation of the sensing CP layer is the most critical issue in the development of MIP-based sensors [128], because the formed polymer should be capable of creating electrostatic interaction, hydrophobic interaction, van der Waals forces, and/or hydrogen bonds between the MIP and analyte molecule [129]. The above-mentioned non-covalent bonds and interactions allow easy binding/dissociation of the imprinted target molecules from the MIP-matrix [103,130,131]. In some MIP formation strategies, functional groups can be attached to polymeric backbone in order to form a complex between the MIP and analyte [104]. Initially imprinted analyte molecules are removed by washing them out from the formed MIP-matrix [102]. In comparison with antibodies or receptors, MIPs have significantly better stability at room temperatures. According to monomers applied in the design of MIPs, there are three general types of such structures: (i) the most simple MIPs are based on one type of monomer, which form CPs [102]; (ii) other types of sensors are based on copolymers, which contain one type of monomer creating a polymer matrix and one or more types of monomers forming a complex with analyte [106]; and (iii) sensors based on ‘overoxidizable’ CPs, which possess functional groups created by electrochemical or chemical overoxidation of polymer, after the entrapment of analyte [110,118].

## 6. Electrochromic Conducting Polymers for Sensor Design

Electrochromism is described as a reversible variation of optical properties of a structure when it is reduced or oxidized by applied electrical current. Materials are considered as electrochromic if they show variations in their visible color. It should be noted that, in some cases, there are more than two oxidation states, and the materials that might exhibit several colors at different oxidation states are called multielectrochromic materials. They can be integrated into optical/electrochemical devices that modulate their optical transmittance, reflectance, absorbance, or light emission. An ideal electrochromic material is expected to have high optic contrast between its extreme states, a short response time, and high stability. In the 21st century, conjugated polymers together with metal oxides and viologens were determined as materials, which are very promising for sensing applications thanks to the easy tailoring of their properties by structural modifications, facile preparation, good processability, and low costs [1]. Exposure of electrochromic polymers to certain gases, vapors, and other analytes results in a change in the electronic structure of the polymer, as observed in their optical spectra, and this is also accompanied by a change of conductivity. In polyaniline (PANI) and polypyrrole (Ppy), as well as in poly(3,4-ethylenedioxythiophene)/poly(styrenesulfonate) (PEDOT/PSS), the electrical conductivity is achieved by creating charge carriers through p-type (hole-based) or n-type (electron donor-based) doping of the conjugated polymer backbone. These conducting polymers (CPs) can be doped during various redox processes, i.e., chemical or electrochemical partial oxidation or reduction. The doping/de-doping processes mentioned above results in reversible or irreversible changes of electrical and optical properties of these conducting polymers and their composites. CPs such as PANI and Ppy and their composites, as well as PEDOT/PSS, are attractive for the design of electrochromic sensors. Very successful applications of these CPs as elements in electrochromic sensors have been demonstrated.

The electrochromic properties of some conducting polymers offer multi-mode-based options for the registration of analytical signal, because such sensors could be based on the combination of various electrochemical and optical techniques. This direction of analytical chemistry emerged very recently, and it has high potential to be applied in various fields of analytical and bioanalytical chemistry. Some aspects of electrochromic polyaniline deposition and the application of conducting polymers for optical sensing systems of Cu(II) ions [132], as well as for some gaseous materials (e.g., CO_2_ and NH_3_), which are able to change the pH of solution where electrochromic sensor electrode was applied [133,134,135], were reported. Optical sensors based on polyaniline exhibit sensitivity and the ability to operate at ambient conditions.

Recently, various optically active materials have been applied as signal transducers in the design of various analytical and bio-analytical systems [136,137]. Conducting polymers [24,62,138] have recently been among the most interesting optical materials, and can offer electrochemical [9], optical [132], and some other properties, which makes them well suited for sensor and biosensor design. A variety of conducting polymer synthesis techniques have been developed. Some very simple synthesis protocols have been reported by our research group, e.g., basic chemical and easily degradable agents such as hydrogen peroxide can be applied for the synthesis of conducting polymer (polypyrrole) [139], polyaniline [37,140], polythiophene [38], polyphenanthroline [9], nanobiocomposite based on poly(1,10-phenanthroline-5,6-dione), poly(pyrrole-2-carboxylic acid) [141], poly-9,10-phenanthrenequinone [39], carbazole [142], azobenzene [143], and some other conducting polymers. Some conducting polymers possess low cost, good environmental stability, and tunable properties [5], and they can form a number of various composites with organic [144], inorganic [145], and biological materials [145], which broadens the range of applicability for such composite materials based on the conducting polymers. Some of above mentioned optically active conducting polymers (e.g., polypyrrole, polyaniline) well serve as immobilization matrixes for biological recognition elements [146,147]; therefore, they have been used as transducers of sensors [148]. It is well known that proper doping of conducting polymer usually results in the increase of electric conductivity by several orders of magnitude [149].

In addition to this, optical properties including electrochromic properties of conducting polymers also significantly depend on different factors including proper doping of the CP-based layer [150]; therefore, this opens up the prospects for the application of optical sensing platforms for the detection and quantification of some analytes.

## 7. Biocompatibility-Related Aspects of Some Conducting Polymers

Biocompatibility-related aspects of conducting polymers are very important, because some biosensors and biofuel cells have recently been implanted in the body [151,152] or attached to the skin [153] of the patient. Therefore, the biocompatibility of these devices is a very important issue; otherwise, non-biocompatible structures can cause inflammation and/or other serious disorders [154,155]. Our investigations showed rather good biocompatibility of the conducting polymer, polypyrrole, towards different ‘biological systems’. Ppy particles induced merely a non-significant irritation to the mammalian immune system [30] and Ppy nanoparticles minimally affected the viability of steam cells [28,29]. Particularly in previous research, it was reported that Ppy does not show any significant toxicity towards mice peritoneum cells, and no changes in immune-related hematological parameters were observed [30]. However, some toxicity of Ppy nanoparticles towards bone marrow-derived stem cells was determined [28]. However, the toxicity observed towards stem cells was dose-dependent and, at lower concentrations, Ppy did not show any toxicity towards primary mouse embryonic fibroblasts (MEFs), mouse hepatoma cell line (MH-22A), and human T lymphocyte Jurkat cells [29]. Hence, our investigations showed that some conducting polymers possess rather good biocompatibility with mammalian stem cells [28,29] and do not irritate mices’ immune system when they are inserted into mice peritoneum [30]. In addition, some other researchers determined that electrical stimulation promotes nerve cell differentiation on the polypyrrole/poly(2-methoxy-5 aniline sulfonic acid) composite [156]. The biocompatibility of conducting polymer polyaniline was also investigated and confirmed [157]. However, the number of similar investigations is still very limited. Advanced biocompatibility of CP-based hydrogels is expected owing to the presence of a large amount of water and some other biocompatible materials (e.g., chitosan [158]) that are usually applied in the formation of the gel; such hydrogels can be applied in various technological fields because of these well applicable mechanical properties [82,159,160]. Some CP-based gels were applied as 3D scaffolds for the accommodation of living cells [161,162] or even as ‘cell delivery carriers’, which are required for transplantation [163] and for some other biomedical applications [164,165,166,167]. Promising biocompatibility aspects of some conducting polymers (e.g., Ppy) encourage one to apply these polymers in the design of biofuel cells [46], which can serve as power sources of some implantable biomedical devices.

However, most researchers are reporting the biocompatibility of CPs based on the possibility to retain the catalytic activity of entrapped enzymes; however, they are not addressing any investigations with living organisms or even with some cell lines. Therefore, experiments on living organisms or at least with some cell lines are still pending and should be performed in order to determine the biocompatibility of most conducting polymers.

## 8. Conclusions

Conducting polymers (CPs) and their composites with various materials have attracted the attention of many researchers; therefore, CPs are applied in the design of sensors and biosensors because of numerous technological advantages they offer. One such advantage is based on various possibilities for the immobilization of biological recognition elements, which are based on enzymes, single stranded DNA (ssDNA), antibodies (Ab), receptors, and/or some other biological recognition exhibiting proteins. Some of these sensors based on conducting polymers offer very high sensitivity, a short response time, and operate at room temperature. Moreover, some conducting polymers can be applied in the design of molecularly imprinted polymers, which are cheap and, in some cases, they can replace natural recognition elements. The electrochromic properties of conducting polymers can also be well exploited in the design of signal transduction systems of some sensors and biosensors. A variety of synthesis methods applied in the synthesis of conducting polymers offer unique possibilities to tune the physical and chemical properties of formed structures. These properties can also be well changed/tuned using different materials, which can be embedded within the structure of formed CPs. Among various synthesis methods, electrochemical deposition of conducting polymer-based layers seems very promising, because electrochemical formation of CPs can be controlled by the adjustment of the most optimal potential/current profile. Some characteristics of conducting polymer-based sensors (namely sensitivity and linear range) are determined by the thickness, density, and permeability of the CP-based layer. Conducting polymer, polypyrrole (Ppy), is the most frequently used in the design of sensors and biosensors. Enzymatic and microbial synthesis of CPs demonstrated that, in such a way, the formed Ppy-based bio-composite materials are well suited for the development of biosensors and biofuel cells. The applicability of enzymatic methods for the synthesis of other conducting polymers such as polyaniline, polythiophene, and some other conducting or π-π conjugated polymers was confirmed by the studies overviewed here. One of the major challenges in the development of CP-based amperometric biosensors used in continuous measurements is related to the stability of the analytical signal. The CP-based layer swells during continuous measurements and, therefore, the analytical signal changes in time. However, this problem can be easily solved by periodical recalibration of such amperometric biosensors after a certain number of measurements. However, the long-term stability of enzymatic biosensors is still a challenge, which needs additional investigation. Promising biocompatibility aspects of some conducting polymers encourage one to apply these polymers in the design of implantable biofuel cells, which can serve as power sources of some implantable biomedical devices.

## Data Availability

Data available in a publicly accessible repository.
